# Validation of Standard and New Criteria for the Differential Diagnosis of Narrow QRS Tachycardia in Children and Adolescents

**DOI:** 10.1097/MD.0000000000002310

**Published:** 2015-12-28

**Authors:** Karol Deutsch, Sebastian Stec, Piotr Kukla, Aleksandra Morka, Marek Jastrzebski, Artur Baszko, Maciej Pitak, Janusz Sledz, Kamil Fijorek, Mariusz Mazij, Bartosz Ludwik, Marcin Gubaro, Leslaw Szydlowski

**Affiliations:** From the ELMedica EP-Network, Kielce (KD, SS, JS); Medical University of Warsaw, Warsaw (KD, MG); Department of Electroradiology, University of Rzeszow, Rzeszow (SS); PCISN, G.V.M. Carint, Sanok (SS); Department of Internal Disease and Cardiology, Specialistic Hospital, Gorlice (PK); Department of Cardiosurgery et Cardiosurgical Intensive Care Polish-American Children's Hospital, Jagiellonian University Medical College in Krakow, Krakow (AM); First Department of Cardiology and Interventional Electrocardiology, University Hospital, Cracow (MJ); Department of Paediatric Cardiology, Poznan University of Medical Sciences, Poznan (AB); Department of Pediatrics, Polish-American Children's Hospital, Jagiellonian University Medical College, Krakow (MP); Carint-Medica, Cracow (JS); Department of Statistics, Cracow University of Economics, Kracow (KF); Regional Specialist Hospital, Research and Development Centre, Wroclaw (MM, BL); and Medical University of Silesia in Katowice, Poland (LS).

## Abstract

To establish an appropriate treatment strategy and determine if ablation is indicated for patients with narrow QRS complex supraventricular tachycardia (SVT), analysis of a standard 12-lead electrocardiogram (ECG) is required, which can differentiate between the 2 most common mechanisms underlying SVT: atrioventricular nodal reentry tachycardia (AVNRT) and orthodromic atrioventricular reentry tachycardia (OAVRT). Recently, new, highly accurate electrocardiographic criteria for the differential diagnosis of SVT in adults were proposed; however, those criteria have not yet been validated in a pediatric population.

All ECGs were recorded during invasive electrophysiology study of pediatric patients (n = 212; age: 13.2 ± 3.5, range: 1–18; girls: 48%). We assessed the diagnostic value of the 2 new and 7 standard criteria for differentiating AVNRT from OAVRT in a pediatric population.

Two of the standard criteria were found significantly more often in ECGs from the OAVRT group than from the AVNRT group (retrograde *P* waves [63% vs 11%, *P* < 0.001] and ST-segment depression in the II, III, aVF, V1–V6 leads [42% vs 27%; *P* < 0.05]), whereas 1 standard criterion was found significantly more often in ECGs from the AVNRT group than from the OAVRT group (pseudo r′ wave in V1 lead [39% vs 10%, *P* < 0.001]). The remaining 6 criteria did not reach statistical significance for differentiating SVT, and the accuracy of prediction did not exceed 70%. Based on these results, a multivariable decision rule to evaluate differential diagnosis of SVT was performed.

These results indicate that both the standard and new electrocardiographic criteria for discriminating between AVNRT and OAVRT have lower diagnostic values in children and adolescents than in adults. A decision model based on 5 simple clinical and ECG parameters may predict a final diagnosis with better accuracy.

## INTRODUCTION

The primary diagnoses in the differential of regular narrow QRS complex supraventricular tachycardia (SVT) in children and adolescents are atrioventricular nodal reentry tachycardia (AVNRT) and orthodromic atrioventricular reentry tachycardia (OAVRT). Analysis of a standard 12-lead electrocardiogram (ECG) is the first step toward determining treatment strategy, which may include choice of antiarrhythmic drugs, invasive procedure (radiofrequency catheter ablation [CA] vs cryoablation), selection of ablation center, and selection of an interventional cardiologist.^1–8^

In 2009 and 2011, new electrocardiographic criteria for the differential diagnosis of SVT were reported to be highly accurate in adult populations. These criteria, which include the presence of a positive r′ deflection in lead aVR during SVT which is absent during the sinus rhythm (SR) and notch of the QRS complex in lead aVL during SVT but not during the SR have not yet been tested and validated in a pediatric population.^2–3^ The purpose of this multicenter study was to determine the value of the standard and new ECG criteria for the differential diagnosis of SVT in children and adolescents under 18 years of age who already underwent CA.

## MATERIALS AND METHODS

The study included 219 consecutive pediatric patients (including those with AVNRT, OAVRT or atrial tachycardia [AT]) who underwent an electrophysiological study (EPS) and CA between January 2010 and December 2013. Patients provided written informed consent, and a local institutional review board approved the study, which complied with the Declaration of Helsinki. Patient ECGs were recorded at 3 different medical centers and retrospectively evaluated. In addition to the AVNRT and OAVRT groups, 7 ECGs that showed AT with 1 to 1 conduction were included in the evaluation but not in the statistical data. All patients on antiarrhythmic drugs discontinued them at least 5 half-lives prior to their planned EPS and ablation procedure. During EPS, arrhythmia was induced by pacing or medications if it had not occurred spontaneously. Patients with pre-excitation on the ECG during SR as well as those with atrial flutter, atrial fibrillation, and structural heart disease were excluded from the study. The mechanism of SVT was precisely established during EPS and then confirmed by the performance of a successful ablation.

The 12-lead ECGs were recorded during EPS using an electrophysiological recording system (EP-Tracer, CardioTek, Maastricht, The Netherlands and BARD Lab System, Lowell, MA). All ECGs were recorded at a paper speed of 25 or 50 mm/s with a gain of 10 mm/mV. Standard filter settings of 0.5 and 1000 Hz were used. A non-preexcitadat SR or SVT was recorded for at least 10 s or 10 beats at the beginning of every ECG.

**FIGURE 1:**
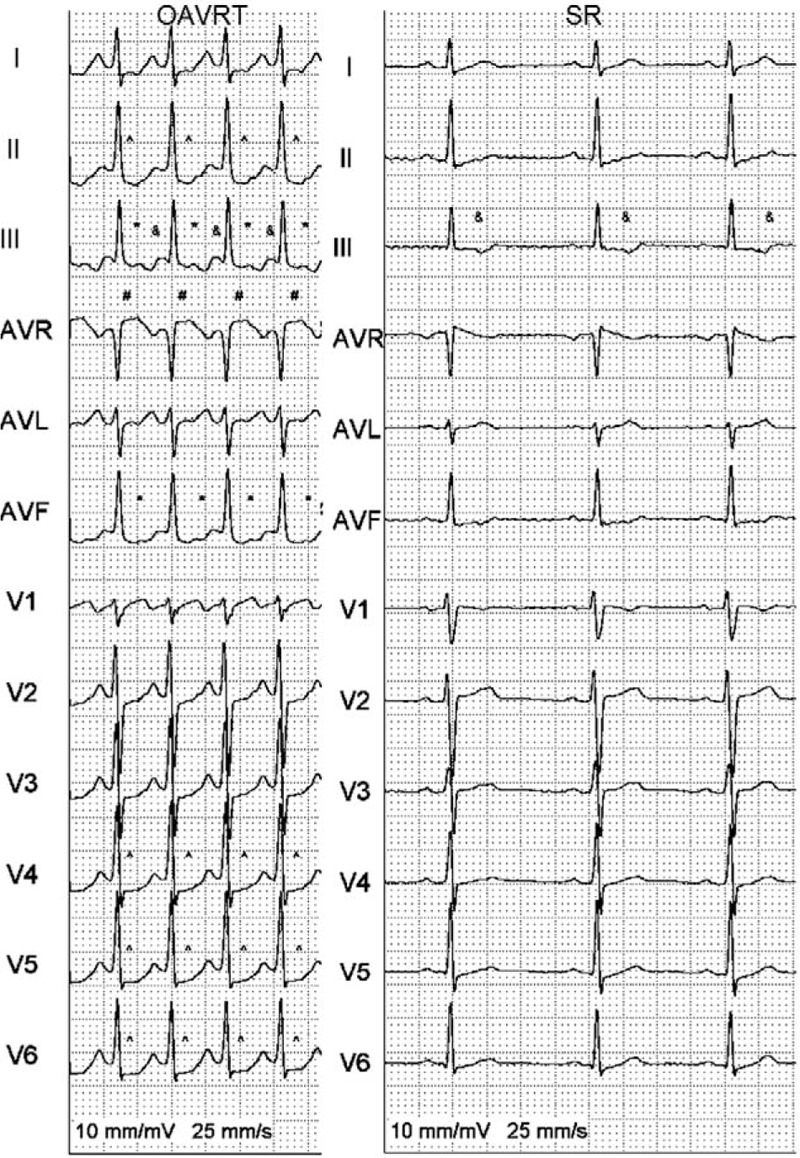
A patient with OAVRT with retrograde P waves in lead III and aVF (^∗^), ST-segment elevation in aVR (#), ST-segment depression in lead II and V4–V5 (^), and T wave inversion in lead III and V1 (&). OAVRT = orthodromic atrioventricular reentry tachycardia, SR = sinus rhythm.

**FIGURE 2:**
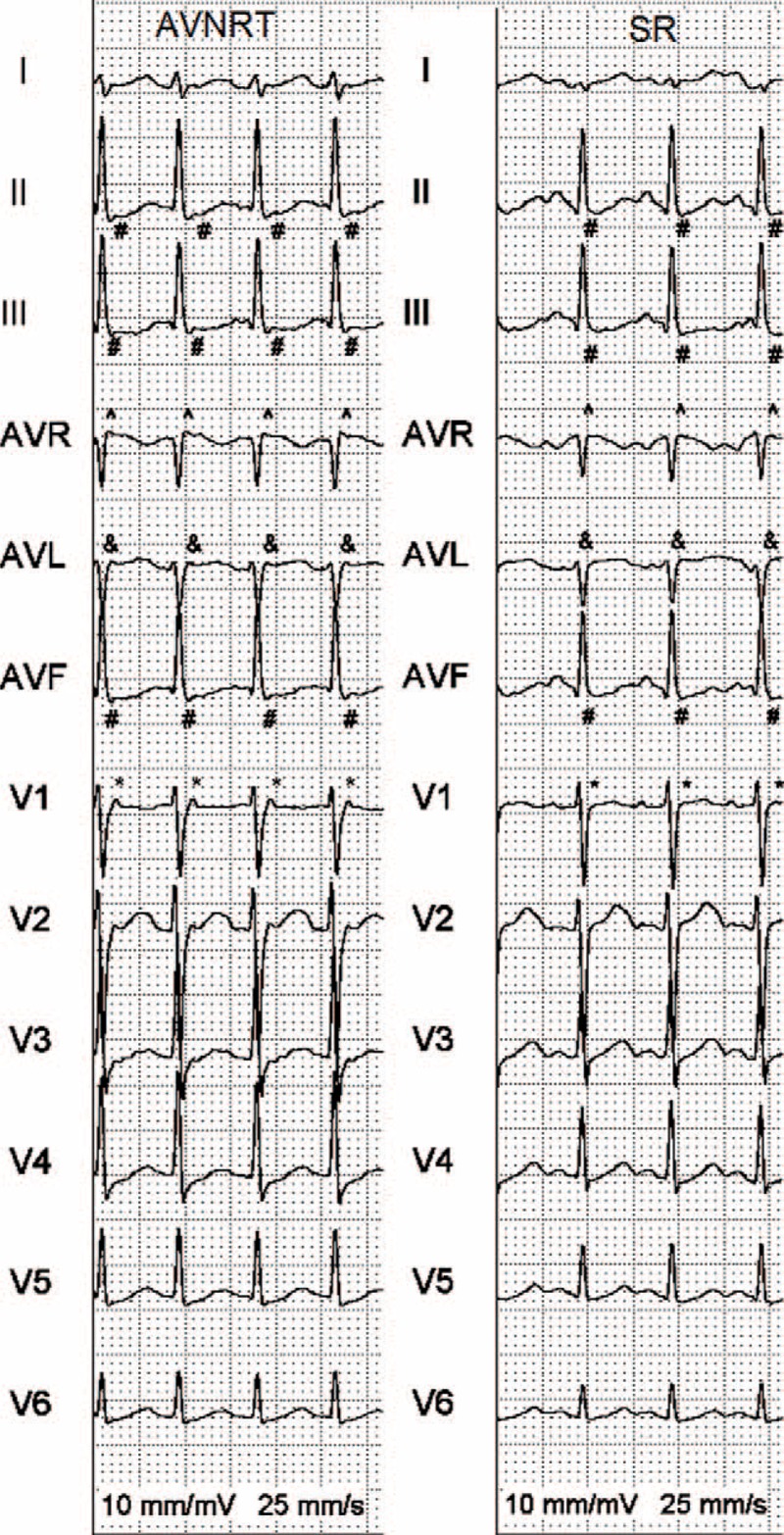
A patient with AVNRT with pseudo r′ wave in V1 (^∗^), r′ wave in aVR (^), aVL notch (&), and the presence of an s wave in sinus rhythm and SVT (#). AVNRT = atrioventricular nodal reentry tachycardia, SR = sinus rhythm.

Each ECG was analyzed for the presence of the standard and new electrocardiographic criteria. The standard criteria were: the presence of retrograde P waves wave in the ST segment in ≥1 lead (Fig. 1); T wave inversion defined as a change in polarization of a T wave for ≥1 mm in ≥1 lead (Fig. 1); QRS alternans defined as a beat-to-beat oscillation in QRS amplitude for ≥1 mm in ≥1 lead during SVT; horizontal or upsloping ST-segment depression ≥2 mm for ≥80 ms at the J point in leads II, III, aVF, or V1–V6 (Fig. 1); horizontal or upsloping ST-segment elevation ≥1 mm at the J point or downsloping ST-segment elevation ≥1.5 mm at the J point for ≥80 ms in lead aVR (Fig. 1); pseudo r′ wave defined as an r′ wave in V1 present during SVT but absent during the SR (Fig. 2); and the presence of pseudo s′ wave defined as an s wave present during SVT but absent during the SR in leads II, III, or aVF (Fig. 2). The new criteria were the presence of a pseudo r′ wave defined as an r′ wave in lead aVR present during SVT but absent during the SR (Fig. 2) and an aVL notch defined as a positive deflection at the end of a QRS complex in lead aVL that is present during SVT but absent during SR (Fig. 2).

The ECGs were assessed by 2 adult cardiologists specialized in arrhythmia management (Sebastian Stec [SMS] and Piotr Kulka [PK]), 2 pediatric cardiologists [Leslaw Szydlowski [LS] and Aleksandra Morka [AM]), and 2 medical students (Karol Deutsch [KD] and Marcin Gubaro [MG]). All researchers were blinded to the mechanism of SVT and the ablation results. The researchers were asked to specify the most likely mechanism of SVT for each ECG based on their clinical judgment. Data from 2 adult cardiologists (SMS and PK) were included into the final evaluation of the diagnostic performance of ECG criteria. Although age and sex were not ECG criteria those data were included in the evaluation as basic patient information.

### Statistical Methods

Continuous variables were presented as means and standard deviations. Categorical variables were presented as counts and percentages. The equality of 2 independent proportions was tested using exact Chi-square test. The performance of binary decision rules was described using diagnostic accuracy, sensitivity, specificity, positive predictive value, negative predictive value, likelihood ratio of a positive test, and likelihood ratio of a negative test (with 95% confidence intervals). The “multivariable decision rule” was developed using logistic regression. Statistical analysis was performed using a “BDTcomparator” program and R 3.0 9,10. *P*-values <0.05 were considered to indicate statistically significant results.

In-depth statistical analysis was performer only on the ECGs of patients with AVNRT and OAVRT as they are the most common mechanisms of SVT in the pediatric population.^9,10^

## RESULTS

Of the 212 ECGs showing SVT, 97 (46%) were due to AVNRT and 115 (54%) were due to OAVRT. Forty-eight percent of the patients were female, and the mean age was 13.2 ± 3.5 years (range 1–18 years). In the AVNRT group, 54% of the patients was female (mean age, 13.6 ± 3.3) and 44% of the patients were female in the OAVRT group (mean age, 12.6 ± 3.8). In both groups gender were not statistically significantly different (*P* = 0.188). In the AT group, 43% of the patients were female (mean age, 14.9 ± 1.5). Mean heart rate (HR) in the OAVRT group was 191 ± 25.9 beats per minute (BPM), in the AVNRT group 189 ± 33.8 BPM and in the AT group 187 ± 49.9 BPM.

Of the 7 standard criteria evaluated for the differential diagnosis of SVT, 1 were found significantly more often in the AVNRT group than in the OAVRT group (pseudo r′ wave in V1) and 2 were found significantly more often in the OAVRT group than in the AVNRT group (retrograde P waves, and ST-segment depression) (Table 2). Retrograde P waves were found in 63% of the patients in the OAVRT group and 11% in the AVNRT group, ST-segment depression was found in 42% of the patients in the OAVRT group and 27% in the AVNRT group, and pseudo r′ wave in V1 were found in 10% of the patients in the OAVRT group and 39% of the AVNRT group. The other standard criteria were not valuable for differentiating between AVNRT and OAVRT in children as they are in adult (Table 1).^4–8^ The number of new criteria found in each group was similar and show no statistical differences. The occurrence of a pseudo s′ wave was similar in the SR and SVT, found in 69% of AVNRT cases and 70% of OAVRT cases. Conversely, an r′ wave in the aVR lead was present in 83% of AVNRT patients and 77% of OAVRT patients in the SR.

**TABLE 2:**
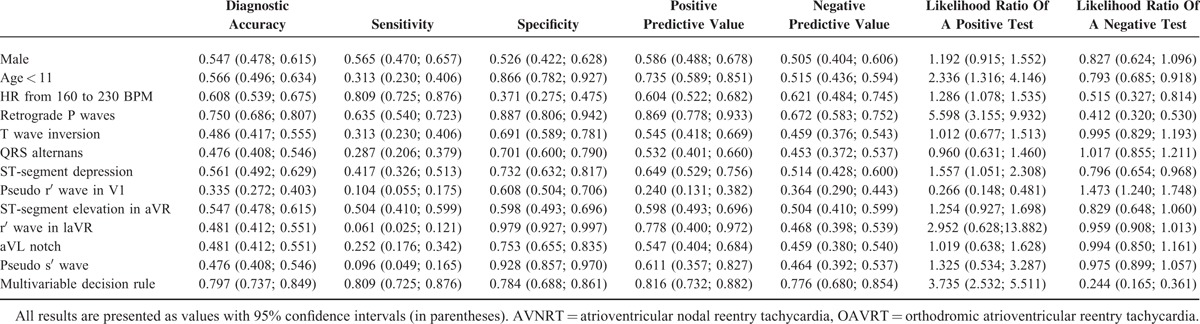
Accuracy, Sensitivity, Specificity, Positive Predictive Value, Negative Predictive Value, Likelihood Ratio of a Positive Test, and Likelihood Ratio of a Negative Test of the Electrocardiographic Criteria in AVNRT Versus OAVRT

**TABLE 1:**
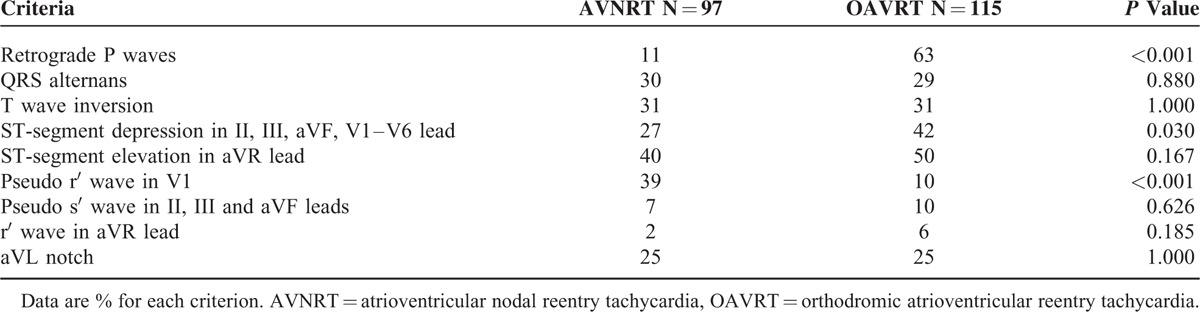
Electrocardiographic Characteristics of the Study Populations

Table 2 shows the diagnostic accuracy, sensitivity, specificity, positive predictive value, negative predictive value, likelihood ratio of a positive test and likelihood ratio of a negative test for all ECG criteria as well as age (age <11 vs age =>11), gender (male vs female), HR (HR between 160 and 230 vs HR below160 or above 230 BPM), and the multivariable decision rule.

Following the univariate analyses the multivariable decision rule (or JUNIOR SVT SCORE) to evaluate the differential diagnosis of SVT was developed. The algorithm uses age, HR, and the presence of: retrograde P waves, ST-segment depression, and pseudo r′ wave in V1 as predictors. Results of more than 1 point indicate OAVRT and results of 1 or less points indicate AVNRT (Table 3). In the study population a result of −2 occurred in nine cases (89% AVNRT, 11% OAVRT), a result −1 occurred in 19 cases (100% AVNRT, 0% OAVRT), a result 0 occurred in 12 cases (83% AVNRT, 17% OAVRT), a result 1 occurred in 53 cases (70% AVNRT, 30% OAVRT), a result 2 occurred in 21 cases (43% AVNRT, 57% OAVRT), a result 3 occurred in 21 cases (33% AVNRT, 67% OAVRT), a result 4 occurred in 45 cases (12% AVNRT, 87% OAVRT), a result 5 occurred in 18 cases (6% AVNRT, 94% OAVRT), a result 6 occurred in 11 cases (0% AVNRT, 100% OAVRT), a result 7 occurred in 3 cases (0% AVNRT, 100% OAVRT).

**TABLE 3:**
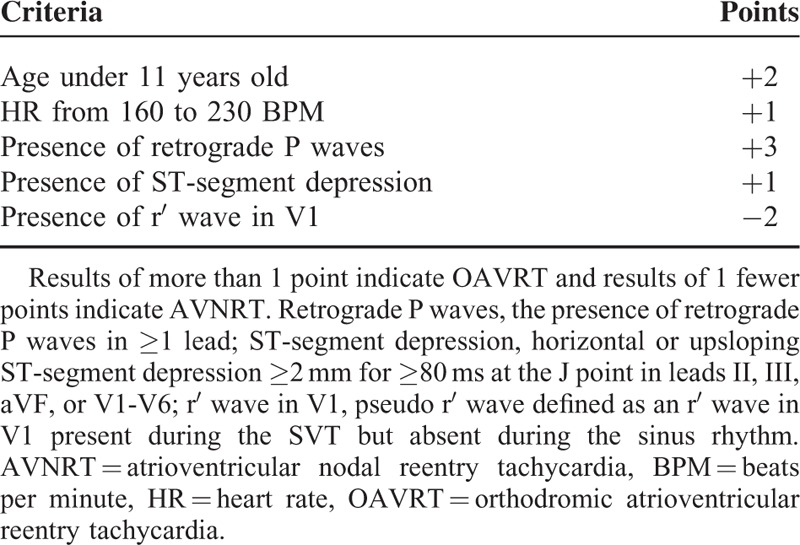
Multivariable Decision Rule (JUNIOR SVT SCORE) to Differentiate Between OAVRT and AVNRT

Among the reviewers, the highest percentage of correct diagnoses of the SVT mechanism based on ECG criteria was approximately 60% in the OAVRT group obtained by SMS, AM and MG, and 73% in the AVNRT group obtained by PK. The rest of the reviewers made correct diagnoses approximately 50% of the time for each group (Table 4). Only 2 reviewers correctly identified AT, but both identified it in only 1 of the 7 cases.

**TABLE 4:**
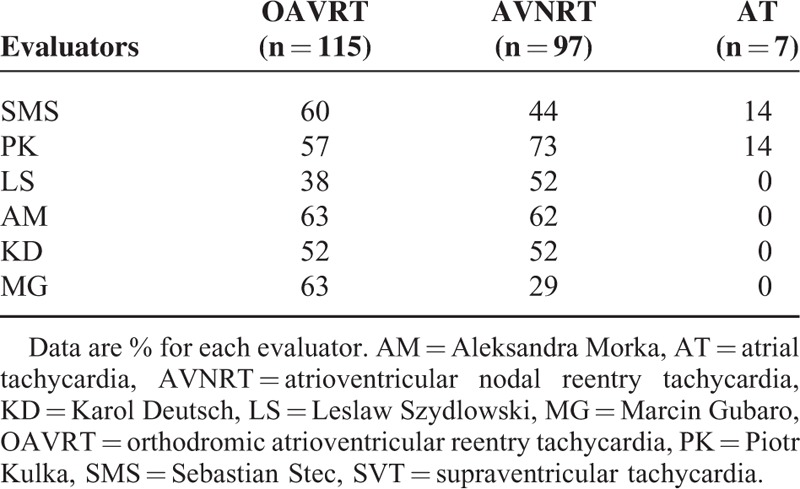
Correct Clinical Diagnosis of SVT, and AT Performed by Each Evaluator

## DISCUSSION

To our knowledge, this is the largest study, in terms of the number of patients and reviewers, to identify the criteria associated with the differential diagnosis of SVT in the pediatric population. Moreover, a clinically useful algorithm was developed to provide practicioners with simple and accurate method for the differential diagnosis of AVNRT and OAVRT.

This study demonstrated that 3 of the 7 standard criteria (retrograde P waves, ST-segment depression, and r′ wave in V1) are valuable for distinguishing AVNRT-associated SVT from OAVRT-associated SVT in a pediatric population. Differences between children and adults such as smaller chest size, right ventricular domination (especially in the youngest patients), fast retrograde activation via the fast pathway, and a smaller distance between chest structures may be responsible for the low value of ECG criteria in children compared to adults.^11^ Using the ECG criteria in the setting of the clinical examination and patient history might increase the odds of correctly diagnosing the mechanism of SVT in children using ECG criteria. In addition, the use of noninvasive methods of ECG monitoring over a prolonged period, such as a Holter monitor or tele-ECG, might increase the odds of making a proper diagnosis.

A previous study of ECG criteria in a pediatric population found that retrograde P waves and ST-segment elevation in aVR were found more frequently in OAVRT, and a pseudo r′ wave in V1 was found mostly in AVNRT. This study also showed the high value of ST-segment depression for the differential diagnosis of SVT, which was not noted in previous studies. These different results may have been due to the larger test group and lower average mean age^1^; however, none of these criteria had the same sensitivity and specificity reported in the adults.^2–8^

The low prevalence of the pseudo s′ wave criteria in this study coincided with a high prevalence of s waves in both the SR and SVT (Figs. 1 and 2). This was not noted in previous studies of this kind in a pediatric population.^1^ Similar findings were seen with the r′ wave in aVR criteria (Fig. 1); however, this is the first study evaluating these criteria in a pediatric population.

Of all the possible standard and new criteria for the differential diagnosis of SVT, only 3 criteria were useful when only the ECG during tachycardia was used to diagnose the mechanism of SVT. These criteria are retrograde P waves, ST-segment elevation in aVR, and ST-segment depression. Most of them were accurate for the identification of OAVRT, and they might be useful in situations when SVT onset is acute in a nonmedical setting.

In both the OAVRT and AVNRT groups, the reviewers in our study missed more diagnoses than in previous adult and pediatric studies.^1,2,6^ Compared to the results of previous studies in pediatric populations, our results might be more authentic due to our study having a larger study population. The missed diagnosis of AT might be because it is less common than AVNRT and OAVRT, and there are no specific 12-lead ECG criteria to identify AT when a 1:1 atrial to ventricular ratio is present.

Recent study in the differential diagnosis of wide QRS tachycardia shows that using a scoring system increases the chance that a reviever will correctly diagnose arrhythmia compared to algorithms systems, suggesting that diagnosis based on our JUNIOR SVT SCORE will be more reliable than previously reported algorithms.^1,12^

Differentiation between AVNRT and OAVRT based on ECG before performing CA might allow electrophisologist to prepare for different approaches, and reduce cost of the additional catheters and resources. Experience with simplified 2-catheter procedure with a non-fluoroscopic approach have reduces the need for lead-apron for medical staff and fluoroscopic exposure to less than 5% of cases with AVNRT.^13^ Transseptal puncture in left-sided OAVRT may require more expertise in retrograde access or transseptal puncture, and some centers will prefer cryoablation for AVNRT.

Our study has several limitations. First, although we investigated the standard and new criteria for the differential diagnosis of SVT, we only investigated 2 of the possible mechanisms of SVT (AVNRT and OAVRT), excluding other mechanisms such as AT due to the low number of cases. Only short episodes of SVT (usually within the first minute of tachycardia induction) recorded in the EP lab was evaluated.

## CONCLUSIONS

Visible P waves in the ST segment, ST-segment depression in II, III, aVF, and V1-V6, as well as pseudo-r′-waves in lead V1 are valuable for the differential diagnosis of SVT by ECG in a pediatric population; however, the new ECG criteria showed only limited diagnostic ability in the pediatric population. Unfortunately, the overall diagnostic ability of all ECG criteria for determining the mechanism of SVT in children and adolescents was very low despite the use of an adult cardiologist, pediatric cardiologist, and medical students as reviewers. The need for a simple, accurate tool to determine the differential diagnosis remains. In addition, an algorithm for the diagnosis of SVT in children and adolescents is yet to be developed. Based on the results of this study, a multivariable decision rule (JUNIOR SVT SCORE) for the differential diagnosis of SVT in a pediatric population will be validated.

## References

[1] Jaeggi ET Gilljam T Bauersfeld U Electrocardiographic differentiation of typical atrioventricular node reentrant tachycardia from atrioventricular reciprocating tachycardia mediated by concealed accessory pathway in children. *Am J Cardiol* 2003; 91:1084–1089. 12714151 10.1016/s0002-9149(03)00153-x

[2] Haghjoo M Bahramali E Sharifkazemi M Value of the aVR lead in differential diagnosis of atrioventricular nodal reentrant tachycardia. *Europace* 2012; 14:1624–1628. 22547768 10.1093/europace/eus109

[3] Di Toro D Hadid C López C Utility of the aVL lead in the electrocardiographic diagnosis of atrioventricular node re-entrant tachycardia. *Europace* 2009; 11:944–948. 19525496 10.1093/europace/eup130

[4] Ho YL Lin LY Lin JL Usefulness of ST-segment elevation in aVR during tachycardia for determining the mechanism of narrow QRS complex tachycardia. *Am J Cardiol* 2003; 92:1424–1428. 14675578 10.1016/j.amjcard.2003.08.051

[5] Kalbfleisch SJ el-Atassi R Calkins H Differentiation of paroxysmal narrow QRS complex tachycardias using the 12-lead electrocardiogram. *J Am Coll Cardiol* 1993; 21:85–89. 8417081 10.1016/0735-1097(93)90720-l

[6] González-Torrecilla E Almendral J Arenal A Combined evaluation of bedside clinical variables and the electrocardiogram for the differential diagnosis of paroxysmal atrioventricular reciprocating tachycardias in patients without pre-excitation. *J Am Coll Cardiol* 2009; 53:2353–2358. 19539146 10.1016/j.jacc.2009.02.059

[7] Letsas KP Weber R Siklody CH Electrocardiographic differentiation of common type atrioventricular nodal reentrant tachycardia from atrioventricular reciprocating tachycardia via a concealed accessory pathway. *Acta Cardiol* 2010; 65:171–176. 20458824 10.2143/AC.65.2.2047050

[8] González-Torrecilla E Almendral J Arenal A Independent predictive accuracy of classical electrocardiographic criteria in the diagnosis of paroxysmal atrioventricular reciprocating tachycardias in patients without pre-excitation. *Europace* 2008; 10:624–628. 18400770 10.1093/europace/eun084

[9] R Core Team, R: A language and environment for statistical computing, R Foundation for Statistical Computing, Vienna, Austria, http://www.R-project.org, 2013, Accessed 2013.

[10] Fijorek K Fijorek D Wisniowska B BDTcomparator: a program for comparing binary classifiers. *Bioinformatics* 2011; 27:3439–3440. 21998157 10.1093/bioinformatics/btr574

[11] Shoei K Huang S Wood Catheter Ablation of Cardiac Arrhythmias. 2011; Philadelphia: Elsevier, 607–633.

[12] Jastrzebski M Sasaki K Kukla P The ventricular tachycardia score: a novel approach to electrocardiographic diagnosis of ventricular tachycardia. *Europace* 2015; [Epub ahead of print]. 10.1093/europace/euv118 25995387

[13] Stec S Sledź J Mazij M Feasibility of implementation of a “simplified, No-X-Ray, no-lead apron, two-catheter approach” for ablation of supraventricular arrhythmias in children and adults. *J Cardiovasc Electrophysiol* 2014; 25:866–874. 24654678 10.1111/jce.12414

