# Validation of Standard and New Criteria for the Differential Diagnosis of Narrow QRS Tachycardia in Children and Adolescents: Erratum

**DOI:** 10.1097/01.md.0000480682.57439.33

**Published:** 2016-02-08

In the article “Validation of Standard and New Criteria for the Differential Diagnosis of Narrow QRS Tachycardia in Children and Adolescents”,^1^ which appeared in Volume 94, Issue 51 of *Medicine*, relevant funding information was originally omitted. Dr. Kukla's name was also misspelled in the text. The article has since been corrected online.
